# The effect of carbapenem-resistant versus carbapenem-susceptible *Enterobacterales* infections on patient outcomes at an academic medical center

**DOI:** 10.1017/ash.2024.445

**Published:** 2024-10-25

**Authors:** Justin A. Clark, David S. Burgess, Daniela C. Moga

**Affiliations:** 1 College of Pharmacy, University of Kentucky, Lexington, KY, USA; 2 College of Public Health, University of Kentucky, Lexington, KY, USA

## Abstract

**Objective:**

We sought to compare patient outcomes between carbapenem-resistant *Enterobacterales* (CRE) and carbapenem-susceptible *Enterobacterales* (CSE) infections at our academic medical center.

**Design:**

We conducted a retrospective cohort study of adult patients with a positive culture of E. coli, E. cloacae, K. aerogenes, K. oxytoca, and/or K. pneumoniae admitted at UK HealthCare (January 1, 2010–December 31, 2019). Based on the type of pathogen on the date of the first culture (index date), patients were included in the CRE (i.e., exposed) group, or the CSE (comparator) group. Exclusion criteria were age < 18 years old, pregnancy, endocarditis, osteomyelitis, necrotizing fasciitis, or cystic fibrosis. We evaluated the impact of CRE vs CSE on a composite outcome of 30-day of all-cause mortality or discharge to hospice using Kaplan–Meier survival curves and Cox proportional hazard regression with inverse probability of treatment weights (IPTW).

**Results:**

Of 17,839 hospitalized patients, 128 and 6,953 patients were included in the CRE and CSE groups, respectively. Baseline differences existed in sex-assigned-at-birth, admission source, time-to-index culture, and infection type/severity. Most CRE index cultures observed (76%) only exhibited resistance to ertapenem. IPTW-adjusted HR [95% CI] of composite outcome was 0.99 [0.65, 1.51] after 30 days. Follow-up analysis in patients with carbapenem-non-susceptible *Enterobacterales*bloodstream infections on index yielded an HR of 1.38 [0.85, 2.24].

**Conclusions:**

Risk of composite outcome was not estimated to differ between patients with CRE and CSE in the overall analysis. Although follow-up analysis identified an increased risk, we cannot statistically distinguish this from a null effect.

## Introduction

Carbapenem-resistant *Enterobacterales* (CRE) infections are a serious global threat. In 2017, the World Health Organization declared CRE to be a critical priority pathogen and emphasized the dire need for drug development and discovery efforts to be focused on these resistant pathogens.^1^ A recent estimation of the global burden of antimicrobial resistance reported that 55,700 deaths were attributable to carbapenem-resistant *K. pneumoniae* alone, with 29,500 and 15,300 more being attributable to carbapenem-resistant *E. coli* and *Enterobacter* species, respectively.^2^ In the United States alone, the most recent Centers for Disease Control and Prevention Threats Report estimated that CRE infections were annually responsible for 1,100 deaths and a healthcare cost of $130 million.^3^

Carbapenem-resistance poses such a threat because these bacteria are additionally resistant to most, if not all, other beta-lactam antimicrobials. Beta-lactams are the backbone of therapy for many Gram-negative infections given their bactericidal activity and high therapeutic index. Critically, cross-resistance to other commonly used antimicrobials, such as fluoroquinolones and aminoglycosides, is often present in carbapenem-resistant bacteria, leaving few viable options remaining. As a result, the time to appropriate therapy for these patients is delayed, which has been shown to increase the likelihood of mortality, especially in the setting of bloodstream infections.^4–6^

This study sought to investigate the impact of CRE infections on patient outcomes at a large academic medical center in a non-endemic region for CRE to further our understanding of these infections.

## Methods

### Study design and patient selection

This was a single-center, retrospective cohort study of adult patients admitted to the University of Kentucky HealthCare between January 1, 2010–December 31, 2019 with a culture-confirmed infection with one of the following five species: *Escherichia coli, Enterobacter cloacae, Klebsiella aerogenes, Klebsiella oxytoca,* or *Klebsiella pneumoniae*. We chose these 5 species specifically as they account for a high percentage of CRE cases.^5^ The study was approved by the University of Kentucky Institutional Review Board. Data were abstracted from the University of Kentucky Center for Clinical and Translational Science Enterprise Data Trust and the clinical microbiology culture and susceptibility database. Data abstracted included patient demographics and hospital admission information, comorbidity data for each admission, and culture information including culture source, culture date, organism identified, and susceptibility data.

The index date was the time of the first culture of one of the above species from a body site consistent with either a bloodstream, intra-abdominal, respiratory, skin/soft tissue, or urinary tract infection during the eligible hospitalization. If any organism isolated during the index culture was resistant to any carbapenem tested, the patient was included in the CRE group; if all organisms isolated were susceptible to all carbapenems tested, the patient was included in the CSE group. Clinical and Laboratory Standards Institute resistant breakpoints for ertapenem, doripenem, imipenem, and meropenem were used (2 mg/mL for ertapenem and 4 mg/mL for the others).^7^

**Figure 1.**
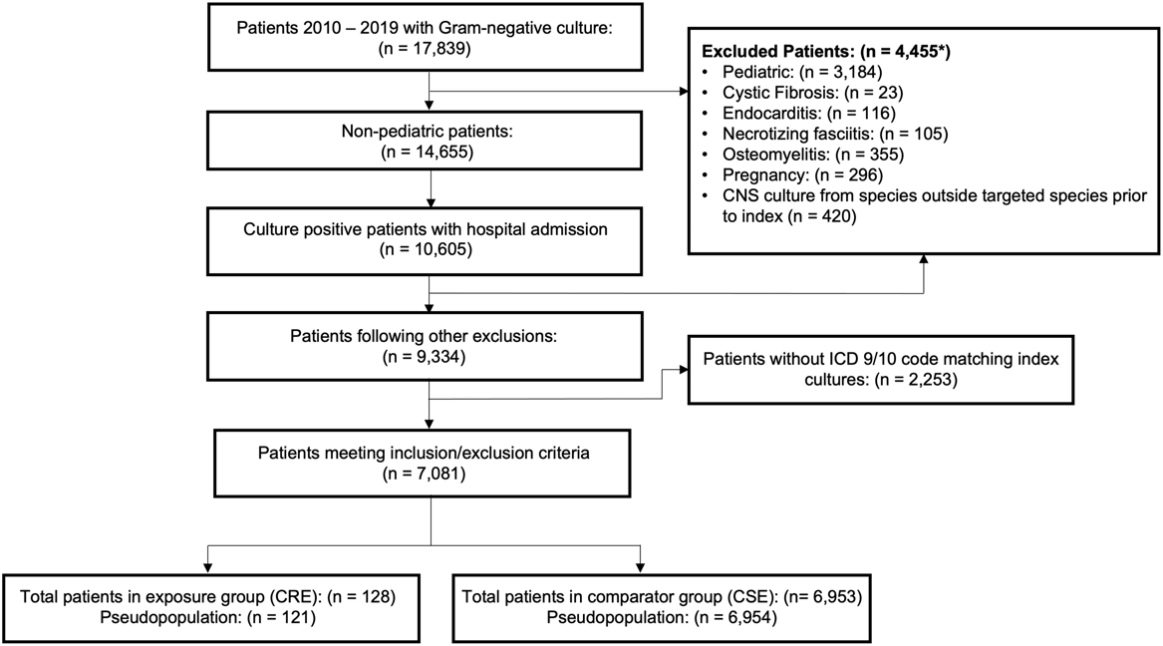
Inclusion and exclusion criteria. Sample selection procedure. *Some patients had multiple exclusion criteria met, so the overall count will be less than the sum of the counts of each variable.

**Figure 2.**
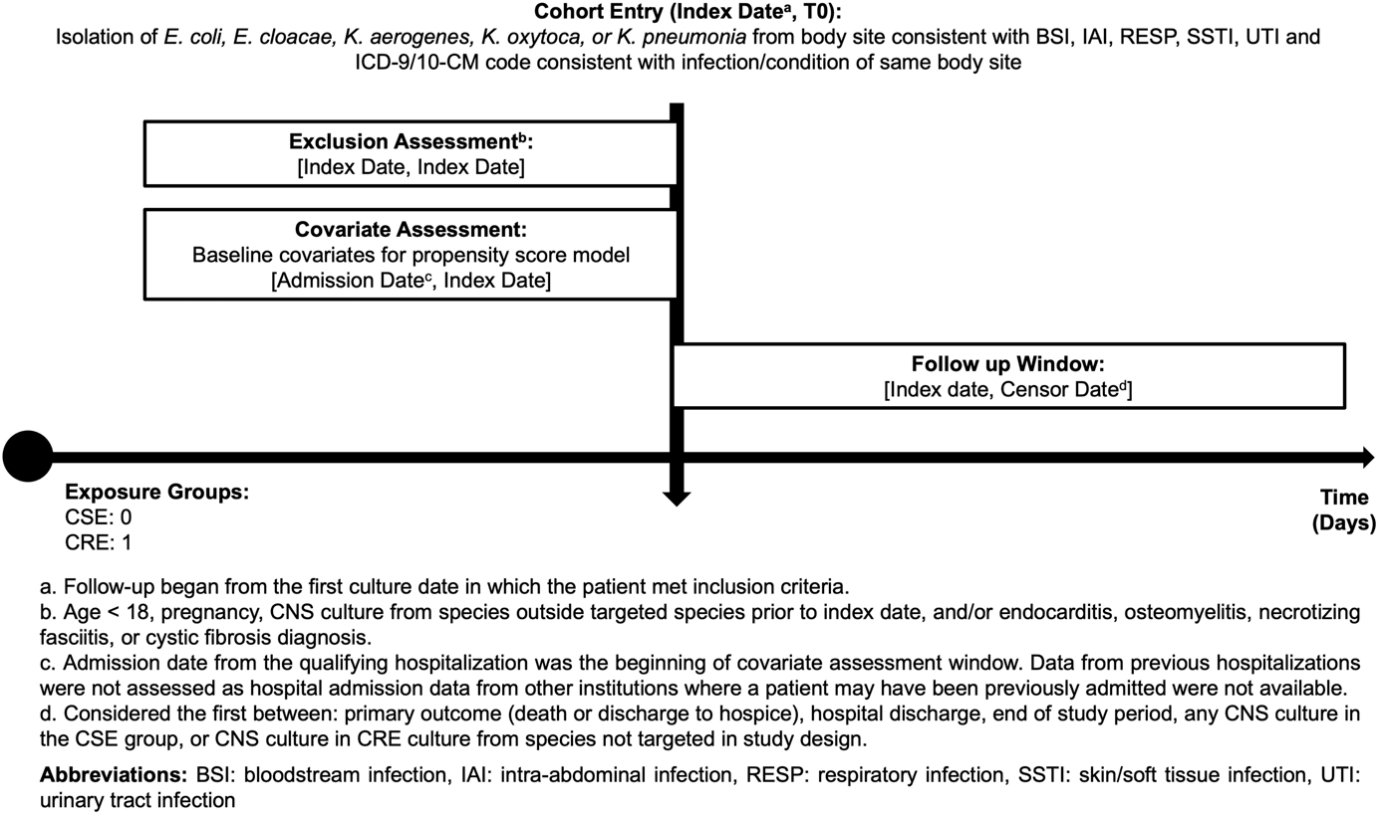
Cohort study design diagram. CRE, carbapenem-resistant *Enterobacterales*; CSE, carbapenem-susceptible *Enterobacterales*; CNS, carbapenem-non-susceptible; BSI, bloodstream infection; IAI, intra-abdominal infection; RESP, respiratory infection; SSTI, skin/soft tissue infection; UTI, urinary tract infection.

Patients met inclusion criteria if one of the index cultures belonged to one of the 5 identified species, the culture was isolated from a body site consistent with either a bloodstream, intra-abdominal, respiratory, skin/soft tissue, or urinary tract infection, and the patient had an International Classification of Diseases (ICD)-9/10-CM code consistent with an infection/condition of the culture site. Patients were excluded if they were <18 years old, pregnant, or had a diagnosis of endocarditis, osteomyelitis, necrotizing fasciitis, or cystic fibrosis during the admission as determined by presence of an ICD-9/10-CM code. Patients were also excluded if they were culture-positive for a carbapenem-non-susceptible, Gram-negative organism not belonging to the 5 targeted species prior to the index date. Supplementary Tables 1 and 2 provide the ICD-9/10-CM codes used to define each criterion. Figures 1 and 2 illustrate the study selection process and cohort study design, respectively. For patients having multiple hospitalizations during the studied timeframe, only the first meeting inclusion/exclusion criteria were considered for analysis.

### Baseline confounding and adjustment

Baseline covariates to address confounding were assessed using electronic health records information available at admission and between the admission date and the index date. Confounders were selected for inclusion using an *a-priori*, theory-based approach and included age at admission, sex assigned at birth, whether the index culture was obtained in the ICU, time to index culture from admission, Charlson Comorbidity Index (CCI) score (modified per Glasheen et al), isolation of a blood or respiratory culture on index, isolation of a lactose-non-fermenting Gram-negative organism on index (ex. *Pseudomonas aeruginosa*), isolation of methicillin-resistant *Staphylococcus aureus* (MRSA) on index, and hospital admission source.^8^ The propensity score for each patient was estimated using logistic regression.^9^ Stabilized weights for inverse probability of treatment weights (IPTW) were calculated using the propensity score as described by Xu et al.^10^ These weights were utilized to generate a “pseudopopulation” of patients with equivalent distributions of the included baseline characteristics wherein the average treatment effect could be estimated. To ensure that balance was achieved between the groups, standardized mean differences (SMDs) were calculated for each of the included covariates in the propensity score model before and after IPTW adjustment. SMD of ≤0.1 was considered to be balanced following IPTW adjustment.^9^

### Data analysis/outcomes

Descriptive statistics for baseline variables were reported as mean (standard deviation (SD)) for parametric variables, median (interquartile range) for non-parametric continuous or interval variables, and counts (%) for categorical variables. We performed summary statistics of routinely tested antimicrobials against Gram-negative bacteria on the isolates collected in the index cultures. All minimum inhibitory concentrations were measured as part of routine clinical practice using either the BD Phoenix^™^ system or E-test gradient strips.

The primary outcome of interest was a composite of all-cause mortality and discharge to hospice assessed at 30 days and secondarily at 14 days following the index date. A Cox proportional hazard model was used to estimate the hazard ratios (HRs) with 95% confidence intervals (CIs) for each outcome. Risks were visually inspected using Kaplan–Meier (KM) curves. In the IPTW-adjusted analysis, the HR 95% CI was estimated using a robust sandwich variance estimator. Furthermore, bootstrapping was used to estimate the 95% CI of IPTW-adjusted KM curves.^11,12^ Follow-up was continued from the date of the index culture until the patient either experienced the composite outcome or was censored (Figure 2). All data analysis was performed using Python (v3.7).

We performed sensitivity analyses to test the robustness of our results to specific assumptions. The first analysis utilized an outcome of all-cause mortality instead of the composite outcome. We also performed the analysis using a broader exposure of carbapenem-non-susceptible *Enterobacterales* (CNSE), which included patients having any index culture in which the isolate was intermediate or resistant to any carbapenem tested. Additionally, we performed a *post-hoc* analysis in a cohort that only included patients having a bloodstream infection at index using the CNSE exposure. A *post-hoc* sensitivity was also performed to determine the effect of time-to-index differences at baseline between the CRE and CSE groups on the composite outcome. This was necessary because sufficient baseline balance was not achieved in this variable following IPTW adjustment. This analysis stratified patients with index cultures ≤72 hours versus >72 hours.

## Results

### Baseline characteristics

Overall, out of 17,839 culture-positive patients for one of the Gram-negative species of interest, 6,953 were included in the CSE group and 128 in the CRE group following inclusion and exclusion criteria (Figure 1). Baseline comparisons of measured covariates between the CRE and CSE groups may be found in Table 1. Both groups were of similar age and race at baseline with a mean age of ∼60 years old and ∼90% white. The CSE group had a higher prevalence of female patients (59.9%%) than the CRE group (48.4%). More of the CSE patients were admitted from nonhealthcare origins than those in the CRE group (40.1% vs 29.7%), while the CRE group included more patients arriving via transfer from another hospital than the CSE group (51.6% vs 36.4%). Furthermore, CRE patients were more likely to have an index culture from a respiratory source than those in the CSE group (21.9% vs 10.9%), while the opposite was true for urinary source (44.5% vs 57.8%).

**Table 1.** Baseline covariates of patients in CRE and CSE groups

Covariates^a^	Overall (n = 7,081)	CRE (n = 128)	CSE (n = 6,953)
Age: Mean (SD)^†^	60.3 (16.4)	59.5 (15.5)	60.3 (16.4)
Sex assigned at birth: Female^†^	4,224 (59.7%)	62 (48.4%)	4,162 (59.9%)
Race			
American Indian/Alaskan Native	5 (0.1%)	0 (0%)	5 (0.1%)
Black/African American	599 (8.5%)	6 (4.7%)	593 (8.5%)
Hispanic	11 (0.2%)	0 (0%)	11 (0.2%)
Native Hawaiian/Pacific Islander	7 (0.1%)	0 (0%)	7 (0.1%)
Unspecified	70 (1.0%)	2 (1.6%)	68 (1.0%)
White	6,389 (90.2%)	120 (93.8%)	6,269 (90.2%)
Admission Source^†^			
Clinic/physician office	783 (11.1%)	11 (8.6%)	772 (11.1%)
Hospital transfer	2,595 (36.6%)	66 (51.6%)	2,529 (36.4%)
Nonhealthcare origin	2,827 (39.9%)	38 (29.7%)	2,789 (40.1%)
Other health facility	469 (6.6%)	9 (7.0%)	460 (6.6%)
Unspecified	407 (5.7%)	4 (3.1%)	403 (5.8%)
Infection Type at Index^b,‡^			
Bloodstream	1,363 (19.2%)	26 (20.3%)	1,337 (19.2%)
Intra-abdominal	324 (4.6%)	7 (5.5%)	317 (4.6%)
Respiratory	784 (11.1%)	28 (21.9%)	756 (10.9%)
Skin/soft tissue	600 (8.5%)	11 (8.6%)	589 (8.5%)
Urinary Tract	4,074 (57.5%)	57 (44.5%)	4,017 (57.8%)
Index Culture taken in ICU^†^	1,678 (23.7%)	51 (39.8%)	1,627 (23.4%)
Time-to-Index (in days): Median (IQR)^†^	1.0 (0.0, 5.0)	5.5 (0.8, 15.0)	1.0 (0.0, 5.0)
Post Index Length of Stay: Median (IQR)	6.0 (3.0, 13.0)	11.0 (5.0, 20.3)	6.0 (3.0, 13.0)
Lactose-non-fermenting Culture at Index^†^	296 (4.2%)	14 (10.9%)	282 (4.1%)
MRSA Culture at Index^†^	219 (3.1%)	10 (7.8%)	209 (3.0%)
Charlson Comorbidity Index			
CCI Score at Index: Median (IQR)^†^	5.0 (3.0, 7.0)	5.0 (3.0, 7.0)	5.0 (3.0, 7.0)
Myocardial Infarction	1,050 (14.8%)	14 (10.9%)	1,036 (14.9%)
Congestive Heart Failure	1,590 (22.5%)	35 (27.3%)	1,555 (22.4%)
Peripheral Vascular Disease	780 (11.0%)	20 (15.6%)	760 (10.9%)
Cerebral Vascular Disease	877 (12.4%)	19 (14.8%)	858 (12.3%)
Dementia	717 (10.1%)	7 (5.5%)	710 (10.2%)
Chronic Pulmonary Disease	2,049 (28.9%)	48 (37.5%)	2,001 (28.8%)
Rheumatologic Disease	232 (3.3%)	7 (5.5%)	225 (3.2%)
Peptic Ulcer Disease	220 (3.1%)	5 (3.9%)	215 (3.1%)
Mild Liver Disease	1,035 (14.6%)	26 (20.3%)	1,009 (14.5%)
T2DM without Chronic Complications	2,225 (31.4%)	44 (34.4%)	2,181 (31.4%)
T2DM with Chronic Complications	762 (10.8%)	17 (13.3%)	745 (10.7%)
Hemiplegia/Paraplegia	547 (7.7%)	10 (7.8%)	537 (7.7%)
Mild - Moderate Renal Disease	1,295 (18.3%)	28 (21.9%)	1,267 (18.2%)
Severe Renal Disease	445 (6.3%)	18 (14.1%)	427 (6.1%)
Any malignancy^c^	1,115 (15.7%)	15 (11.7%)	1,100 (15.8%)
Moderate-Severe Liver Disease	531 (7.5%)	19 (14.8%)	512 (7.4%)
Metastatic Solid Tumor	564 (8.0%)	5 (3.9%)	559 (8.0%)
HIV	29 (0.4%)	0 (0.0%)	29 (0.4%)

CCI, Charlson Comorbidity Index; CRE, Carbapenem-resistant *Enterobacterales*; CSE, Carbapenem-susceptible *Enterobacterales*; ICU, Intensive care unit; IQR, Interquartile range; MRSA, Methicillin-resistant *Staphylococcus aureus*; SD, Standard deviation; T2DM, Type 2 diabetes mellitus.

a Continuous variables are presented as Mean (SD) if parametric or Median (IQR) if otherwise, and ordinal/nominal variables are presented as N (%)

b Percentages may sum to > 100% because some patients have multiple infection types at index

c Includes lymphoma and leukemia, but excludes malignant non-melanoma neoplasm of skin

† Variable included in the propensity score model

‡ Variable included in the propensity score model as dichotomous variable indicating whether patient had either a bloodstream or respiratory isolate cultured at index or not.

Baseline patient severity also appeared to be higher in the CRE group, having a higher prevalence of index cultures being isolated in an ICU (39.8% vs 23.4%). The CRE group also had a higher likelihood of having a positive culture for either a lactose-non-fermenting species (namely *P. aeruginosa*) or MRSA at index (10.9% vs 4.1%) and (7.8% vs 3.0%), respectively. Patients in the CRE group were more likely to have index cultures collected later in the admission than the CSE group (5.5 [0.8, 15.0] vs 1.0 [0.0, 5.0]). Despite this difference in apparent severity of illness, both groups had a similar prevalence of comorbidities, assessed with the CCI. Following IPTW adjustment, baseline balance was achieved with respect to all covariates included in the propensity score model between the patients in the CRE and CSE groups except for time-to-index culture and, to a lesser degree, admission source. SMD values obtained before and after IPTW adjustment are reported in the supplementary materials.

### Microbiological analysis

**Figure 3.**
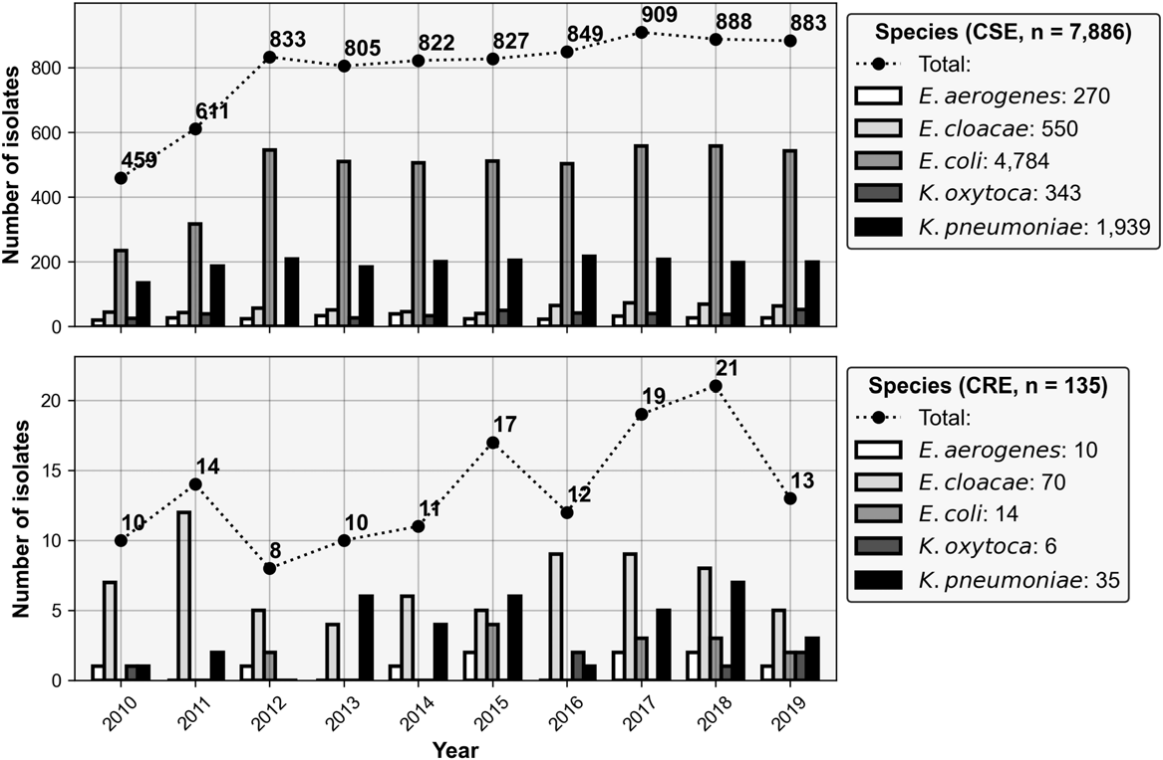
Annual distributions of index cultures for carbapenem-resistant *Enterobacterales* (CRE) and carbapenem-susceptible *Enterobacterales* (CSE) groups by species. Above are the total number of index cultures observed in the CSE and CRE groups (scatter plot) plotted over the counts of the individual species comprising the overall count (bar plot).

The overall incidence of CRE infections within our patient cohort across 10 years was 1.8%. As illustrated in Figure 3, the number of CSE isolates gradually increased over the duration of the study, except for the sharp increase between the years 2010 and 2012 caused by an expansion of our academic medical campus. Most isolates collected in the CSE group were *E. coli* followed by *K. pneumoniae*. The predominant species in the CRE group was *E. cloacae* followed by *K. pneumoniae*; however, the annual distribution of the CRE species was more varied than in the CSE group. Supplementary Figure 1 illustrates the distribution of each culture source by species.

A total of 135 index cultures were isolated from 128 patients in the CRE group and 7,886 from 6,953 patients in the CSE group (Table 2). CRE isolates often demonstrated lowered susceptibilities than the CSE isolates. Gentamicin and tobramycin exhibited considerable drops in susceptibility (92% to 80% and 91% to 77%, respectively), while amikacin susceptibility remained similar. Meropenem was the next most active agent (71%) in the CRE group, exhibiting susceptibilities similar to ciprofloxacin, levofloxacin, and sulfamethoxazole-trimethoprim (62%, 65%, and 68%, respectively). No other beta-lactam demonstrated susceptibilities >20% other than cefepime (42%). The majority of carbapenem resistance was determined by ertapenem resistance alone (n = 103). These isolates often demonstrated similar non-beta-lactam susceptibility to CSE isolates, while isolates resistant to other carbapenems accounted for sharp declines in susceptibilities (Supplementary Table 3).

**Table 2.** Susceptibility analysis of index cultures collected from patients in CRE versus CSE groups

Antimicrobials	CRE (n = 135)						CSE (n = 7,886)					
	n	Min MIC	MIC_50_	MIC_90_	Max MIC	%S	n	Min MIC	MIC_50_	MIC_90_	Max MIC	%S
Amikacin	134	2	8	8	64	99	7,870	1	8	8	64	100
Ampicillin	135	16	32	32	128	0	7,813	4	32	32	512	29
Ampicillin-Sulbactam	115	4	32	32	32	2	7,625	1	8	32	64	53
Aztreonam	130	0.02	32	32	32	19	7,646	0.03	2	2	32	92
Ceftazidime	132	0.5	32	32	32	14	7,801	0.13	0.5	2	32	93
Ceftriaxone	121	0.25	64	64	64	5	4,102	0.02	1	64	128	78
Cefepime	118	0.25	4	32	32	42	7,832	0.03	1	1	32	93
Cefepime (SDD)	118	0.25	4	32	32	65	7,832	0.03	1	1	32	95
Cefoxitin	132	4	32	32	32	8	7,744	4	4	32	512	81
Cefazolin	130	2	32	32	32	2	6,695	1	2	32	32	51
Ciprofloxacin	116	0.5	0.5	4	4	62	7,561	0.06	0.5	4	4	74
Ertapenem	133	0.5	2	8	32	2	7,748	0.004	0.5	0.5	0.5	100
Nitrofurantoin	105	16	128	128	128	28	6,800	2	16	128	128	76
Gentamicin	128	0.5	2	16	16	80	7,752	0.5	2	2	64	92
Levofloxacin	131	0.25	1	8	8	65	7,763	0.03	1	8	64	76
Meropenem	132	0.03	1	8	32	71	7,825	0.02	1	1	1	100
Piperacillin-Tazobactam	130	2	128	512	512	12	7,680	1	2	16	512	94
Sulfamethoxazole-Trimethoprim	135	0.25	0.5	4	4	68	7,771	0.06	0.5	4	64	77
Tetracycline	127	2	2	16	16	65	7,722	2	2	16	512	76
Tobramycin	134	1	2	16	16	77	7,803	0.13	2	2	64	91

CLSI susceptibility breakpoints were utilized for all antimicrobials (7). Of note, we used breakpoints of 2 and 1 μg/mL for levofloxacin and ciprofloxacin, which differ from the M100 30^th^ edition cited.

%S, percent susceptible; MIC_n_, MIC necessary for inhibiting n^th^ percent of isolates tested; SDD, Susceptible-dose-dependent; CRE, Carbapenem-resistant *Enterobacterales*; CSE, Carbapenem-susceptible *Enterobacterales.*

*Ceftriaxone was not routinely assessed for Gram-negative cultures throughout the entire study duration

### Survival analysis

**Figure 4.**
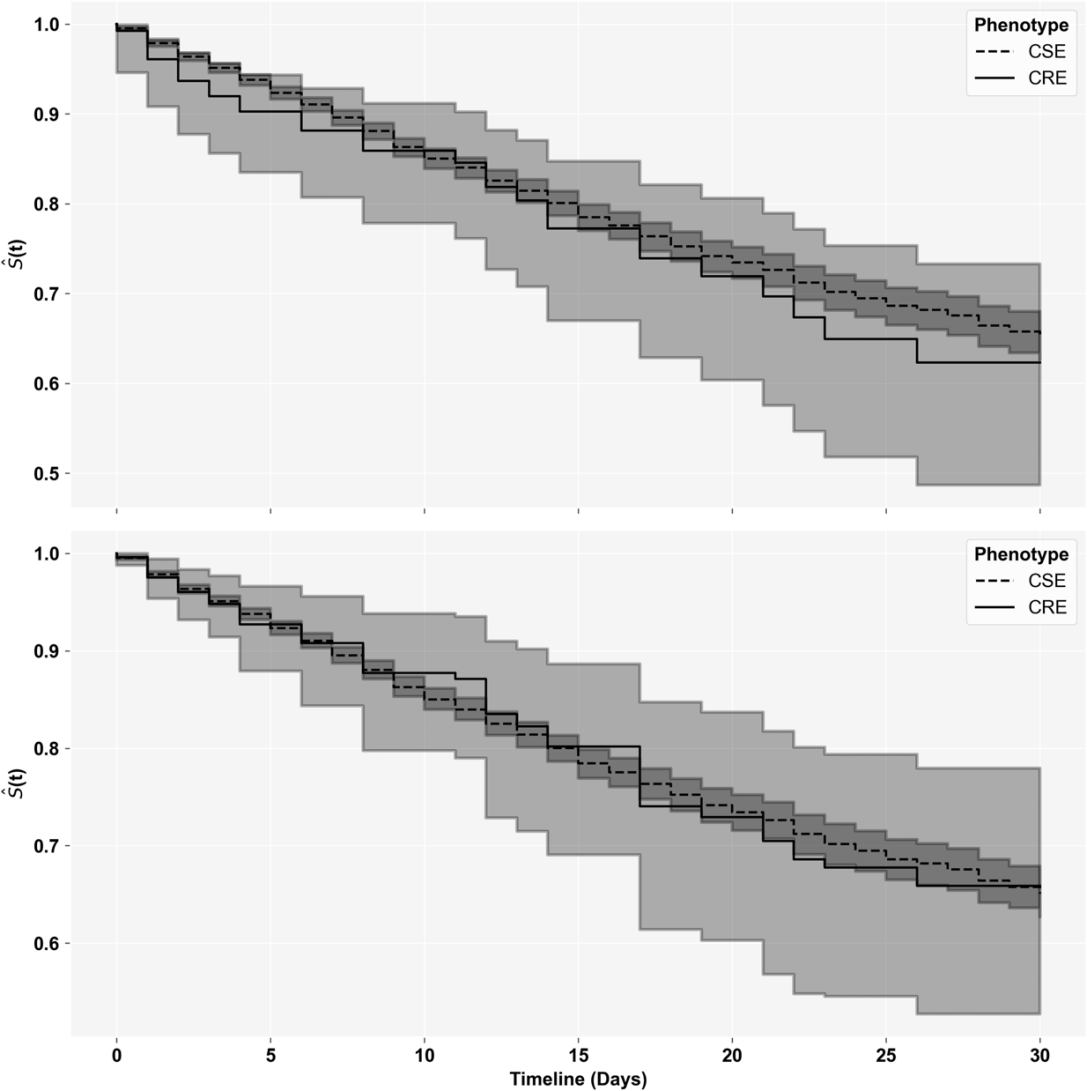
Kaplan–Meier Curves of 30-day composite outcome. Above are Kaplan–Meier Survival Curves comparing (top) crude and (bottom) IPTW-adjusted 30-day Composite Outcome between the carbapenem-resistant *Enterobacterales* and carbapenem-susceptible *Enterobacterales* groups.

The analysis of 14- and 30-day composite outcome between the CRE and CSE groups is presented in Table 3. Overall, 29 composite outcomes and 1,679 patient days in the CRE group and 996 outcomes and 64,628 patient days in the CSE group were observed within 30 days of follow-up, with most events occurring within 14 days of follow-up. This corresponds to a crude HR [95% CI] of 1.14 [0.79, 1.65] and IPTW-adjusted HR [95% CI] of 0.99 [0.65, 1.51] after 30 days. The 14-day composite outcomes were similar to the 30-day outcomes. Crude and IPTW-adjusted KM curves for 30-day follow-up are illustrated in Figure 4.

**Table 3.** Composite outcome assessment of CRE versus CSE infections

	CRE (n = 128)	CSE (n = 6,953)		HR [95% CI]
14-day Composite Outcome				
# observed events (mortality, hospice)	22 (14, 8)	816 (520, 296)	Crude	1.18 [0.77, 1.8]
Patient Follow-up (Patient days)	1,180	51,192	IPTW	0.98 [0.61, 1.57]
30-day Composite Outcome				
# observed events (mortality, hospice)	29 (18, 11)	999 (634, 365)	Crude	1.14 [0.79, 1.65]
Patient Follow-up (Patient days)	1,679	64,751	IPTW	0.99 [0.65, 1.51]

Composite outcomes included either all-cause mortality or discharge to hospice at the specified follow-up. Hazard ratios and 95% confidence intervals were estimated using Cox proportional hazard regression.

HR, Hazard Ratio; IPTW, inverse probability of treatment weight; CRE, Carbapenem-resistant *Enterobacterales*; CSE, Carbapenem-susceptible *Enterobacterales*.

When performing the analysis using the outcome of all-cause mortality, the crude 14- and 30-day estimates of HR remained unchanged; while the IPTW-adjusted estimates remained in the same direction but were often smaller in magnitude. When using an exposure of CNSE, we observed a crude HR [95% CI] of 1.25 [0.95, 1.64] and IPTW-adjusted HR [95% CI] of 1.02 [0.75, 1.4] after 30 days. When only including patients having a bloodstream infection on index, the respective findings were 1.56 [1.05, 2.31] and 1.38 [0.85, 2.24]. Again, 14-day outcomes were highly similar. In the time-to-index sensitivity analysis, the HRs [95% CI] for patients with index cultures ≤72 hours versus >72 hours were 1.33 [0.69, 2.57] and 1.06 [0.61, 1.85] after 14 days and 1.53 [0.9, 2.61] and 0.92 [0.55, 1.54] after 30 days. These analyses are included in the supplementary materials.

## Discussion

Over the course of a decade at an academic medical center, we did not observe conclusive evidence of an increased risk of 14- or 30-day composite outcome between patients having CRE versus CSE infections. This remained true when expanding the exposure definition to include CNSE. These findings are surprising given that previous studies have consistently reported elevated risk of mortality in patients having CRE versus CSE infections.^13,14^

When only considering patients having bloodstream infections on index using an exposure of CNSE, we noted increased HR following IPTW-adjustment after 14- and 30-day follow-up compared to the analysis including all infection types (Supplementary Tables 9). While these results are also not statistically significant, they may suggest that the large proportion of patients with non-bloodstream infections, particularly the high proportion of urinary cultures, led to an inconsistent overall exposure effect and biased the results towards the null. Furthermore, cultured pathogens may have constituted a colonization event rather than a “true infection”. Even though we implemented measures to avoid misclassification of colonization as infection (ICD-9/10-CM codes), it is doubtful we were able to completely mitigate this issue. This misclassification would also likely bias our estimates towards the null following confounding adjustment. Restricting analysis to patients with bloodstream infections provides a more consistent exposure and prevents this misclassification bias, which has led several studies to investigate only patients with bloodstream infections.^15–18^ One drawback to this approach observed in our study was the limited sample size.

Another noteworthy finding is the predominance of cultures defined as CRE based solely on resistance to ertapenem (76%). Given the high percentage of “mono-resistance” to ertapenem, the elevated susceptibility to meropenem, and the high prevalence of *Enterobacter* species (59%) among the CRE isolates, it also follows that our CRE population is largely driven by a non-carbapenemase-producing (NCP)-CRE phenotype, although the extent to which this is the case is difficult to determine without having performed specific testing for carbapenemase production in our analysis.^19^ Distinguishing carbapenemase production has become increasingly important following the Center for Disease Control and Prevention (CDC) CRE definition change in 2015, which was implemented to improve sensitivity for identifying carbapenemase-producing-CRE (CP-CRE). As a result, NCP-CRE have been increasingly identified, particularly those that are resistant to ertapenem and no other carbapenem.^18–22^

These phenotypic distinctions are highly relevant when contemplating implementation of infection control interventions as these carbapenem resistance phenotypes are mediated by distinctly different mechanisms. For example, carbapenemases are usually carried on a mobile genetic element, which facilitates horizontal spread and transmissibility. NCP-CRE, on the other hand, has not been frequently associated with outbreaks and is largely thought to arise due to accumulated antibiotic exposure.^23,24^ This difference likely implies that a universally applied set of infection control measures will not be a reasonable approach to address all CRE infections; however, more research is needed to specifically elucidate the most effective strategies for each CRE phenotype.

Also impacted by CRE phenotype is the selection of appropriate antimicrobial therapy. The expression of a carbapenemase effectively prevents the use of any carbapenem and requires utilizing a novel antimicrobial agent or combination therapy to appropriately eradicate the infection. NCP-CRE can often be treated with a different carbapenem, such as meropenem or imipenem, due to ertapenem resistance alone defining the CRE phenotype. In the present study in which most isolates are likely NCP-CRE, 71% of all the CRE index cultures were susceptible to meropenem. Antibiotic selection in the treatment of CRE is highly nuanced, and we would recommend the reader utilize more complete resources for guidance in this process as this is not a primary focus of the present study.^25^

A strength of this study is the use of a “new-user” design, which strives to emulate a prospective trial through causal analysis of observational data.^26^ By utilizing IPTW adjustment, we were able to provide effect measure estimates in a balanced population conditional on the confounding variables. Within this design, confounders and independent risk factors for experiencing the outcome were specified *a priori* from subject-matter expertise rather than statistical associations present within the data, as recommended by Hernan et al.^27^ We also performed extensive sensitivity and follow-up analyses to better understand the effect of key assumptions upon our findings. Lastly, our study specifically reported whether our CRE isolates expressed an ertapenem mono-resistance phenotype, which should help further contextualize our results and highlight a phenotype of growing interest.

Limited sample size in the exposure group was insufficiently large to allow more precise estimates, and the single-center nature limits externality of our findings. As previously discussed, defining a “true infection” remains difficult to do in studies that lack expert chart review. Further study is needed to accurately distinguish active infections retrospectively with high specificity and sensitivity. As mentioned before, in the overall analysis, baseline balance with respect to time-to-index was not sufficiently achieved (SMD = 0.2) following IPTW adjustment. However, the stratified sensitivity analysis demonstrated no excess risk of composite outcome in the CRE group with index cultures >72 hours after 14- and 30-day follow-up. Furthermore, in the bloodstream infection analysis, much better baseline balance was achieved with respect to time-to-index (SMD = 0.115), which indicates that this variable likely did not unduly bias the analysis. Lastly, due to our cohort study being retrospective in design, there remains a possibility for unmeasured or residual confounding affecting the results.

CRE infections remain a critical threat to healthcare. Our study sought to estimate the impact of these infections on patient outcomes compared to susceptible infections but was unable to provide conclusive evidence. Follow-up analyses were suggestive of an effect among patients with bloodstream infections caused by CRE or CNSE, but even after a 10-year study timeline, we lacked sufficient precision to make any definitive claim. This study highlights the importance of multicenter collaboration in the study of CRE infections, as they will likely be necessary to observe an appropriately sized patient population. Future and present endeavors must continue to investigate best practices for the rapid identification and treatment of both CP-CRE and NCP-CRE in the ongoing struggle with these massive threats.

## Supporting information

Clark et al. supplementary material Clark et al. supplementary material

## Supplementary material

For supplementary material accompanying this paper visit http://doi.org/10.1017/ash.2024.445. click here to view supplementary material
