# Supplementary material for: The effect of carbapenem-resistant versus carbapenem-susceptible Enterobacterales infections on patient outcomes at an academic medical center
Source: Antimicrob Steward Healthc Epidemiol. 2024 Oct 25;4(1):e189. doi: 10.1017/ash.2024.445 (PMC11504081; doi:10.1017/ash.2024.445)
Supplement: Clark et al. supplementary material [file S2732494X24004455sup001.docx]

**Supplementary Materials**

**Table of Contents**

[Inclusion Criteria 2](#_Toc149143870)

[Exclusion Criteria 4](#_Toc149143871)

[CRE vs. CSE Breakdown of Index Culture Sources by Species 5](#_Toc149143872)

[MIC Analyses 6](#_Toc149143873)

[Kaplan Meier Curves 8](#_Toc149143874)

[14-day Composite Outcome between CRE vs. CSE in Main Analysis 8](#_Toc149143875)

[14- and 30-day Composite Outcome between CNSE vs. CSE in Main Analysis 9](#_Toc149143876)

[14- and 30-day Composite Outcome between CNSE vs. CSE in Bloodstream Infection Cohort 10](#_Toc149143877)

[Sensitivity Analysis: Composite Outcome vs. All-cause Mortality as Primary Outcome 12](#_Toc149143878)

[Sensitivity Analysis: Time-to-index 13](#_Toc149143879)

[Composite Outcome Summaries of Follow-up Analyses 14](#_Toc149143880)

[Composite Outcome Assessment of CNSE vs. CSE Infections 14](#_Toc149143881)

[Composite Outcome Assessment of CRE vs. CSE Infections in Patients with Bloodstream Infections on the Index Date 15](#_Toc149143882)

[Composite Outcome Assessment of CNSE vs. CSE Infections in Patients with Bloodstream Infections on the Index Date 16](#_Toc149143883)

[Baseline Balance of Covariates Included in the Propensity Score Before and After IPTW-adjustment 17](#_Toc149143884)

[CRE vs. CSE Overall Analysis 17](#_Toc149143885)

[CNSE vs. CSE Main Overall Analysis 19](#_Toc149143886)

[CNSE vs. CSE Bloodstream Infections on Index Date 21](#_Toc149143887)

# Inclusion Criteria

| **Supplementary Table 1.** ICD-9/10 Codes for Inclusion Criteria | | |
| --- | --- | --- |
|  | **ICD-9-CM** | **ICD-10-CM** |
| **Blood Stream Infection** | 038.40, 038.42, 038.49, 038.8, 038.9, 422.92, 790.7, 995.91, 995.92, 996.62, 999.31 | A41.150, A41.151, A41.159, A41.89, A41.9, R65.2x, R78.81, T80.211, T80.218, T80.219, T81.44x |
| **Intra-abdominal Infection** | 531.x, 532.x, 533.1x, 533.2x, 533.5x, 533.6x, 534.1x, 534.2x, 534.5x, 534.6x, 539.01, 539.81, 540.0x, 540.1x, 541, 542, 562.01, 562.03, 562.11, 562.13, 567, 569.5, 569.61, 569.81-569.83, 572.0, 575.0, 575.4, 575.5, 576.1, 576.3, 577.0 | A04.4, A04.8, A04.9, K25.x, K26.x, K27.1, K27.2, K27.5, K27.6, K28.1, K28.2, K28.5, K28.6, K35.2x, K35.3x, K57.0x, K57.2x, K57.4x, K57.8x, K63.0-K63.2, K65.0-K65.2, K65.8, K65.9, K67, K68.11, K68.12, K68.19, K68.9, K81.0, K82.A2, K83.0x, K83.2, K85.02, K85.12, K85.22, K85.32, K85.82, K85.92, K94.02, K94.12, K94.22, K95.01, K95.81 |
| **Respiratory Infection** | 482.0, 482.82, 482.83, 482.89, 482.9, 483.8, 484.8, 485, 486, 510, 513, 997.3 | J15.0, J15.5, J15.6, J15.8, J15.9, J16.8, J17, J18.x, J85.x, J86.x, J95.851 |
| **Skin/Soft Tissue Infection** | 035, 611.0x, 771.5x, 68x.x, 704.8x, 707.x, 910.1, 910.3, 910.5, 910.7, 910.9, 911.1, 911.3, 911.5, 911.7, 911.9, 912.1, 912.3, 912.5, 912.7, 912.9, 913.1, 913.3, 913.5, 913.7, 913.9, 914.1, 914.3, 914.5, 914.7, 914.9, 915.1, 915.3, 915.5, 915.7, 915.9, 916.1, 916.3, 916.5, 916.7, 916.9, 917.1, 917.3, 917.5, 917.7, 917.9, 919.1, 919.3, 919.5, 919.7, 919.9, 998.83, 999.34, 86.0, 86.2 | A46, N61.1, L01.x-L08.x**, L72.8, L72.9, L76, L88, L89, L92.8, L97.x, L98.0x, L98.4x, T80.212x, T81.41x, T81.42x, |
| **Urinary Tract Infection** | 580.x-583.x, 590.x, 595.x*, 597.x, 598.0, 598.5, 598.9, 599.0, 599.2, 599.3, 996.64, 996.65 | N10.x, N11.0, N11.8, N11.9, N12, N13.5, N13.6, N15.1, N28.84-N28.86, N30.x*, N34.0, N34.2, N34.3, N39.0 |
| Included are the ICD-9/10-CM codes utilized when assessing agreement with the index cultures from respective body sites. The included codes directly codify infection or a condition of the cultured areas.  *Except for irradiation cystitis, **Except for Staph scalded skin syndrome | | |

# Exclusion Criteria

| **Supplementary Table 2.** ICD-9/10-CM Codes for Exclusion Criteria | | |
| --- | --- | --- |
|  | **ICD-9-CM** | **ICD-10-CM** |
| **Cystic Fibrosis** | 277.0x | E84.x |
| **Endocarditis** | 421.0, 421.9 | I33.0, I33.9 |
| **Necrotizing Fasciitis** | 728.86 | M72.6 |
| **Osteomyelitis** | 730.x | M86.x |
| **Pregnancy** | 63x.x-679.x, V22.x, V23.x | O0x.x-O7x.x, O80.x, O82.x, O9x.x, Z3x.x |
| Included are ICD-9/10-CM codes used to assess the presence of the respective exclusion criteria. | | |

# CRE vs. CSE Breakdown of Index Culture Sources by Species

| 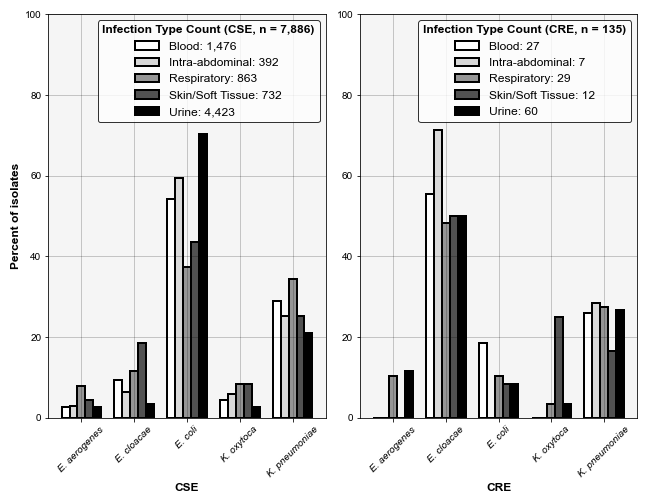 |
| --- |
| **Supplementary Figure 1.** Breakdown of Index Culture Sources by Species  Shown above are the relative percentage each species contributes to the infection types included in the study between the CSE and CRE groups. |

#

# MIC Analyses

| **Supplementary Table 3.** Susceptibility Analysis of Index Cultures collected from Patients in CRE group Stratified on Carbapenem Resistant Phenotype | | | | | | | | | | | | |
| --- | --- | --- | --- | --- | --- | --- | --- | --- | --- | --- | --- | --- |
| **Antimicrobials** | **CRE resistant ≥ 1 anti-pseudomonal carbapenem**  **(n = 32)** | | | | | | **CRE resistant only to Ertapenem**  **(n = 103)** | | | | | |
|  | **n** | **Min**  **MIC** | **MIC_50_** | **MIC_90_** | **Max**  **MIC** | **%S** | **n** | **Min**  **MIC** | **MIC_50_** | **MIC_90_** | **Max**  **MIC** | **%S** |
| **Amikacin** | 31 | 8 | 8 | 16 | 64 | 97 | 103 | 2 | 8 | 8 | 8 | 100 |
| **Ampicillin** | 32 | 16 | 32 | 32 | 32 | 0 | 103 | 16 | 32 | 32 | 128 | 0 |
| **Ampicillin-Sulbactam** | 29 | 8 | 32 | 32 | 32 | 3 | 86 | 4 | 32 | 32 | 32 | 1 |
| **Aztreonam** | 31 | 2 | 32 | 32 | 32 | 19 | 99 | 0.02 | 32 | 32 | 32 | 19 |
| **Ceftazidime** | 31 | 0.5 | 32 | 32 | 32 | 13 | 101 | 0.5 | 32 | 32 | 32 | 14 |
| **Ceftriaxone** | 28 | 1 | 64 | 64 | 64 | 4 | 93 | 0.25 | 64 | 64 | 64 | 5 |
| **Cefepime** | 30 | 1 | 32 | 32 | 32 | 13 | 88 | 0.25 | 2 | 32 | 32 | 51 |
| **Cefepime (SDD)** | 30 | 1 | 32 | 32 | 32 | 23 | 88 | 0.25 | 2 | 32 | 32 | 80 |
| **Cefoxitin** | 31 | 4 | 32 | 32 | 32 | 19 | 101 | 4 | 32 | 32 | 32 | 5 |
| **Cefazolin** | 30 | 4 | 32 | 32 | 32 | 0 | 100 | 2 | 32 | 32 | 32 | 2 |
| **Ciprofloxacin** | 29 | 0.5 | 4 | 4 | 4 | 48 | 87 | 0.5 | 0.5 | 4 | 4 | 67 |
| **Ertapenem** | 30 | 0.5 | 2 | 8 | 8 | 10 | 103 | 2 | 2 | 8 | 32 | 0 |
| **Nitrofurantoin** | 26 | 16 | 128 | 128 | 128 | 31 | 79 | 16 | 128 | 128 | 128 | 27 |
| **Gentamicin** | 28 | 2 | 2 | 16 | 16 | 54 | 100 | 0.5 | 2 | 16 | 16 | 88 |
| **Levofloxacin** | 32 | 1 | 1 | 8 | 8 | 53 | 99 | 0.25 | 1 | 8 | 8 | 69 |
| **Meropenem** | 31* | 1 | 8 | 16 | 32 | 3^**^ | 101 | 0.03 | 1 | 1 | 2 | 92 |
| **Piperacillin-Tazobactam** | 32 | 2 | 128 | 512 | 512 | 16 | 98 | 2 | 128 | 512 | 512 | 10 |
| **Sulfamethoxazole-Trimethoprim** | 32 | 0.5 | 2 | 4 | 4 | 53 | 103 | 0.25 | 0.5 | 4 | 4 | 73 |
| **Tetracycline** | 30 | 2 | 2 | 16 | 16 | 70 | 97 | 2 | 4 | 16 | 16 | 63 |
| **Tobramycin** | 32 | 2 | 4 | 16 | 16 | 53 | 102 | 1 | 2 | 8 | 16 | 84 |
| **Abbreviations:** %S: percent susceptible, MIC_n_: MIC necessary for inhibiting n^th^ percent of isolates tested, SDD: Susceptible-dose-dependent  CLSI susceptibility breakpoints were utilized for all antimicrobials (7). Of note, we used breakpoints of 2 and 1 μg/mL for levofloxacin and ciprofloxacin, which differ from the M100 30^th^ edition cited.  *Meropenem was not tested in one isolate which was resistant to doripenem.  **One isolate included was susceptible to meropenem but resistant to imipenem. | | | | | | | | | | | | |

| **Supplementary Table 4.** Susceptibility Analysis of Index Cultures collected from Patients in CRE vs. non-CRE CNSE in Main Analysis of CNSE vs. CSE | | | | | | | | | | | | |
| --- | --- | --- | --- | --- | --- | --- | --- | --- | --- | --- | --- | --- |
| **Antimicrobials** | **CRE (n = 135)** | | | | | | **Non-CRE CNSE (n = 100)** | | | | | |
|  | n | Min  MIC | MIC_50_ | MIC_90_ | Max  MIC | %S | n | Min  MIC | MIC_50_ | MIC_90_ | Max  MIC | %S |
| Amikacin | 134 | 2 | 8 | 8 | 64 | 99 | 100 | 8 | 8 | 8 | 32 | 99 |
| Ampicillin | 135 | 16 | 32 | 32 | 128 | 0 | 100 | 4 | 32 | 64 | 64 | 6 |
| Ampicillin-Sulbactam | 115 | 4 | 32 | 32 | 32 | 2 | 85 | 4 | 32 | 32 | 64 | 15 |
| Aztreonam | 130 | 0.02 | 32 | 32 | 32 | 19 | 92 | 2 | 32 | 32 | 64 | 9 |
| Ceftazidime | 132 | 0.5 | 32 | 32 | 32 | 14 | 96 | 0.5 | 32 | 32 | 32 | 39 |
| Ceftriaxone | 121 | 0.25 | 64 | 64 | 64 | 5 | 74 | 1 | 64 | 64 | 64 | 5 |
| Cefepime | 118 | 0.25 | 4 | 32 | 32 | 42 | 89 | 1 | 4 | 32 | 32 | 36 |
| Cefepime (SDD) | 118 | 0.25 | 4 | 32 | 32 | 65 | 89 | 1 | 4 | 32 | 32 | 89 |
| Cefoxitin | 132 | 4 | 32 | 32 | 32 | 8 | 94 | 4 | 32 | 32 | 32 | 28 |
| Cefazolin | 130 | 2 | 32 | 32 | 32 | 2 | 95 | 2 | 32 | 32 | 64 | 1 |
| Ciprofloxacin | 116 | 0.5 | 0.5 | 4 | 4 | 62 | 88 | 0.5 | 0.5 | 8 | 8 | 76 |
| Ertapenem | 133 | 0.5 | 2 | 8 | 32 | 2 | 93 | 0.5 | 1 | 1 | 1 | 33 |
| Nitrofurantoin | 105 | 16 | 128 | 128 | 128 | 28 | 87 | 16 | 64 | 128 | 256 | 41 |
| Gentamicin | 128 | 0.5 | 2 | 16 | 16 | 80 | 100 | 2 | 2 | 16 | 32 | 88 |
| Levofloxacin | 131 | 0.25 | 1 | 8 | 8 | 65 | 99 | 1 | 1 | 8 | 16 | 80 |
| Meropenem | 132 | 0.03 | 1 | 8 | 32 | 71 | 100 | 0.03 | 1 | 2 | 2 | 62 |
| Piperacillin-Tazobactam | 130 | 2 | 128 | 512 | 512 | 12 | 100 | 4 | 64 | 512 | 512 | 40 |
| Sulfamethoxazole-Trimethoprim | 135 | 0.25 | 0.5 | 4 | 4 | 68 | 100 | 0.5 | 0.5 | 8 | 512 | 55 |
| Tetracycline | 127 | 2 | 2 | 16 | 16 | 65 | 94 | 2 | 2 | 16 | 16 | 85 |
| Tobramycin | 134 | 1 | 2 | 16 | 16 | 77 | 100 | 2 | 2 | 8 | 32 | 86 |
| **Abbreviations:** %S: percent susceptible, MIC_n_: MIC necessary for inhibiting n^th^ percent of isolates tested, SDD: Susceptible-dose-dependent  CLSI susceptibility breakpoints were utilized for all antimicrobials (7). Of note, we used breakpoints of 2 and 1 μg/mL for levofloxacin and ciprofloxacin, which differ from the M100 30^th^ edition cited. | | | | | | | | | | | | |

# Kaplan Meier Curves

## 14-day Composite Outcome between CRE vs. CSE in Main Analysis

**B.**

**A.**

| 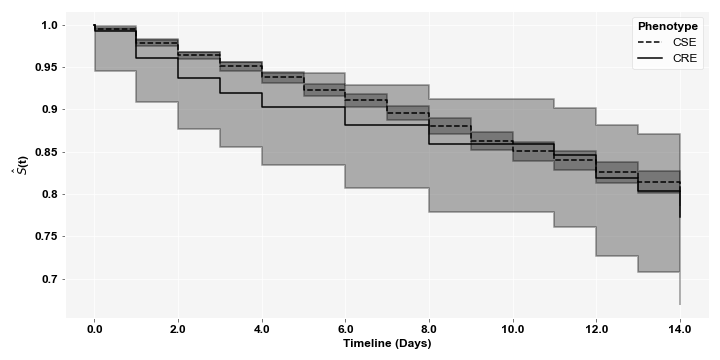 |
| --- |
| 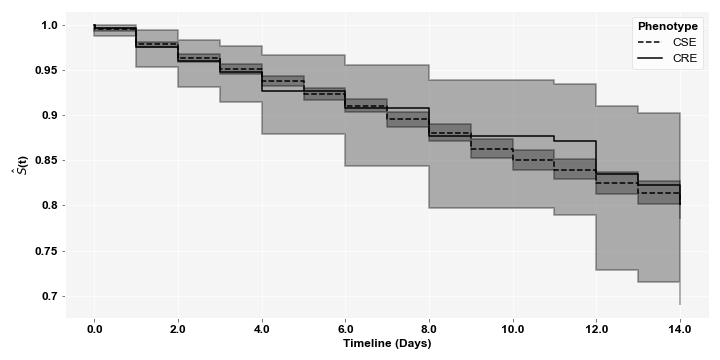 |
| **Supplementary Figure 2.** Kaplan Meier Survival Curve Comparing (A) crude and (B) IPTW-adjusted 14-day Composite Outcome between CRE and CSE Infections |

## 14- and 30-day Composite Outcome between CNSE vs. CSE in Main Analysis

Because the estimates produced from the CNSE vs. CSE Main Analyses were sufficiently similar to the CRE vs. CSE Main Analysis (differences only observed in the precision of 95% CI), these Kaplan Meier curves were not included in the supplementary materials.

## 14- and 30-day Composite Outcome between CNSE vs. CSE in Bloodstream Infection Cohort

| 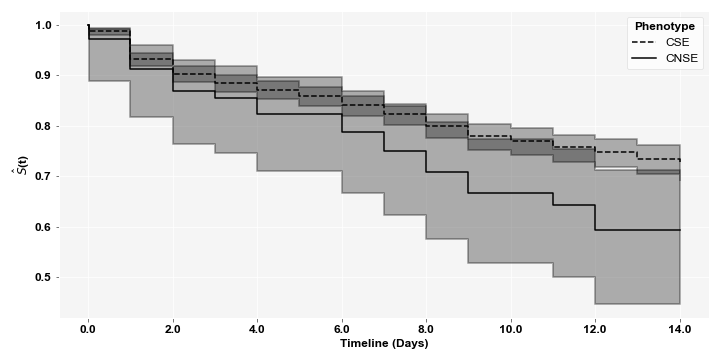  **A.**  **B.** |
| --- |
| 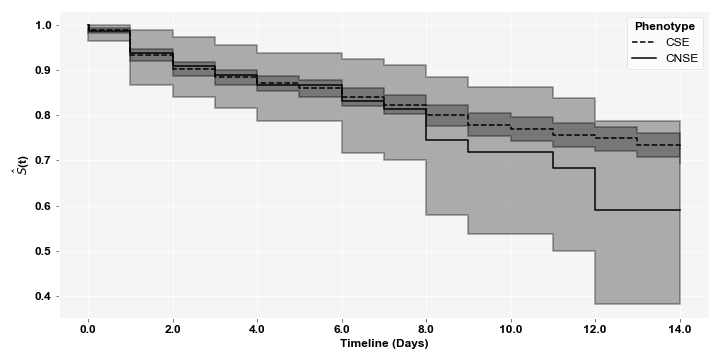 |
| **Supplementary Figure 3.** Kaplan Meier Survival Curve Comparing (A) crude and (B) IPTW-adjusted 14-day Composite Outcome between CNSE and CSE Bloodstream Infections |

| 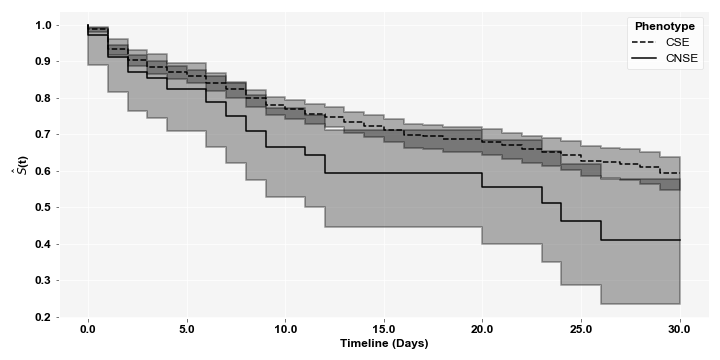  **B.**  **A.** |
| --- |
| 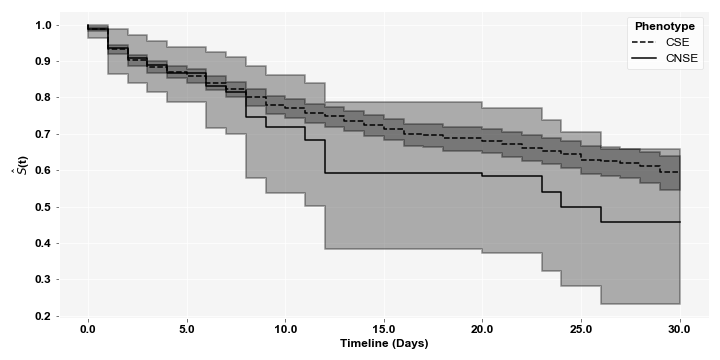 |
| **Supplementary Figure 4.** Kaplan Meier Survival Curve Comparing (A) crude and (B) IPTW-adjusted 30-day Composite Outcome between CNSE and CSE Bloodstream Infections |

**A.**

**B.**

# Sensitivity Analysis: Composite Outcome vs. All-cause Mortality as Primary Outcome

| **Supplementary Table 5.** Hazard Ratio of Outcome of Interest after 14- and 30-day Follow-up | | | | | | | | |
| --- | --- | --- | --- | --- | --- | --- | --- | --- |
|  | **CNSE vs. CSE** | | | | **CRE vs. CSE** | | | |
|  | **14-day Outcome** | | **30-day Outcome** | | **14-day Outcome** | | **30-day Outcome** | |
|  | Crude | IPTW | Crude | IPTW | Crude | IPTW | Crude | IPTW |
| **1** | 1.21  [0.88, 1.66] | 0.95  [0.66, 1.36] | 1.25  [0.95, 1.64] | 1.02  [0.75, 1.4] | 1.18  [0.77, 1.8] | 0.98  [0.61, 1.57] | 1.14  [0.79, 1.65] | 0.99  [0.65, 1.51] |
| **2** | 1.17  [0.77, 1.76] | 0.8  [0.5, 1.26] | 1.28  [0.91, 1.79] | 0.91  [0.61, 1.35] | 1.21  [0.71, 2.06] | 0.88  [0.5, 1.55] | 1.14  [0.72, 1.83] | 0.84  [0.5, 1.42] |
| Row 1 utilized the composite outcome of all-cause mortality and discharge to hospice. Row 2 utilized all-cause mortality as the outcome of interest. All reported values are the HR [95% CI]. | | | | | | | | |

# Sensitivity Analysis: Time-to-index

| **Supplementary Table 6.** Hazard Ratio of 14- and 30-day Composite Outcome in CRE vs. CSE Stratified by Time-to-Index | | |
| --- | --- | --- |
|  | **14-Day**  **Composite Outcome** | **30-Day**  **Composite Outcome** |
| **T ≤ 72 hours** | 1.33  [0.69, 2.57] | 1.53  [0.9, 2.61] |
| **T > 72 hours** | 1.06  [0.61, 1.85] | 0.92  [0.55, 1.54] |
| All reported values are HR [95% CI]; T: time-to-index | | |

# Composite Outcome Summaries of Follow-up Analyses

## Composite Outcome Assessment of CNSE vs. CSE Infections

| **Supplementary Table 7.** Composite Outcome Assessment of CNSE vs. CSE Infections | | | | |
| --- | --- | --- | --- | --- |
| **14-day Composite Outcome** | | | | |
|  | **CNSE**  **(n = 228)** | **CSE**  **(n = 6,946)** |  | HR  [95% CI] |
| # observed events  (mortality, hospice) | 40  (24, 16) | 816  (520, 296) | Crude | 1.21  [0.88, 1.66] |
| Patient Follow-up  (Patient days) | 2,094 | 51,152 | IPTW | 0.95  [0.66, 1.36] |
| **30-day Composite Outcome** | | | | |
| # observed events  (mortality, hospice) | 55  (35, 20) | 999  (634, 365) | Crude | 1.25  [0.95, 1.64] |
| Patient Follow-up  (Patient days) | 2,904 | 64,695 | IPTW | 1.02  [0.75, 1.4] |
| **Abbreviations:** HR: Hazard Ratio, IPTW: inverse probability of treatment weight  Composite outcomes included either all-cause mortality or discharge to hospice at the specified follow-up. Hazard ratios and 95% confidence intervals were estimated using Cox proportional hazard regression. | | | | |

## Composite Outcome Assessment of CRE vs. CSE Infections in Patients with Bloodstream Infections on the Index Date

| **Supplementary Table 8.** Composite Outcome Assessment of CRE vs. CSE Infections in a Subgroup of Patients with Bloodstream Infections on the Index Date in the Main Analysis of CRE vs. CSE | | | | |
| --- | --- | --- | --- | --- |
| **14-day Composite Outcome** | | | | |
|  | **CRE**  **(n = 26)** | **CSE**  **(n = 1,337)** |  | **HR**  **[95% CI]** |
| # observed events  (mortality, hospice) | 11  (9, 2) | 276  (196, 80) | Crude | 2.05  [1.12, 3.75] |
| Patient Follow-up  (Patient days) | 211 | 10,435 | IPTW | -- |
| **30-day Composite Outcome** | | | | |
| # observed events  (mortality, hospice) | 13  (11, 2) | 311  (216, 95) | Crude | 1.99  [1.14, 3.47] |
| Patient Follow-up  (Patient days) | 311 | 13,405 | IPTW | -- |
| **Abbreviations:** HR: Hazard Ratio, IPTW: inverse probability of treatment weight  Composite outcomes included either all-cause mortality or discharge to hospice at the specified follow-up. Hazard ratios and 95% confidence intervals were estimated using Cox proportional hazard regression. IPTW adjustment for this subgroup analysis was not performed due to lack of sufficient sample size in the CRE group. | | | | |

## Composite Outcome Assessment of CNSE vs. CSE Infections in Patients with Bloodstream Infections on the Index Date

| **Supplementary Table 9.** Composite Outcome Assessment of CNSE vs. CSE Infections in Patients with Bloodstream Infections on the Index Date | | | | |
| --- | --- | --- | --- | --- |
| **14-day Composite Outcome** | | | | |
|  | **CNSE**  **(n = 69)** | **CSE**  **(n = 1,405)** |  | **HR**  **[95% CI]** |
| # observed events  (mortality, hospice) | 23  (15, 8) | 289  (205, 84) | Crude | 1.56  [1.02, 2.38] |
| Patient Follow-up  (Patient days) | 577 | 10,964 | IPTW | 1.42  [0.85, 2.36] |
| **30-day Composite Outcome** | | | | |
| # observed events  (mortality, hospice) | 27  (19, 8) | 327  (226, 101) | Crude | 1.56  [1.05, 2.31] |
| Patient Follow-up  (Patient days) | 788 | 14,070 | IPTW | 1.38  [0.85, 2.24] |
| **Abbreviations:** HR: Hazard Ratio, IPTW: inverse probability of treatment weight  Composite outcomes included either all-cause mortality or discharge to hospice at the specified follow-up. Hazard ratios and 95% confidence intervals were estimated using Cox proportional hazard regression. | | | | |

# Baseline Balance of Covariates Included in the Propensity Score Before and After IPTW-adjustment

## CRE vs. CSE Overall Analysis

| **Supplementary Table 10.** Baseline Balance of Covariates included in Propensity Score Model Before and After IPTW Adjustment for CRE vs. CSE Overall Analysis | | |
| --- | --- | --- |
| **Variable** | **Unadjusted SMD** | **Adjusted SMD** |
| Age (years) | -0.055 | 0.084 |
| Gender | 0.231 | 0.077 |
| Index Culture in ICU | 0.359 | 0.016 |
| Time to Index Culture | 0.449 | 0.201 |
| CCI Score | 0.129 | 0.085 |
| Lactose-non-fermenting Culture on Index | 0.264 | 0.080 |
| MRSA culture on Index | 0.214 | 0.051 |
| Blood/Respiratory Culture at Index | 0.255 | 0.044 |
| Admission Source | 0.330 | 0.127 |
| **Abbreviations:** CCI: Charlson Comorbidity Index, IPTW: inverse probability treatment weight, MRSA: methicillin-resistant *Staphylococcus aureus*, SMD: standardized mean difference, ICU: intensive care unit.  The standardized mean differences above were measured before and after the application of IPTW-adjustment to ensure balance on the included covariates was achieved. A value of ≤ 0.1 was considered to be sufficiently balanced. | | |

| **Supplementary Figure 5.** Standardized Mean Difference of Covariates included in the Propensity Score Model Before and After IPTW Adjustment for CRE vs. CSE Overall Analysis |
| --- |
| 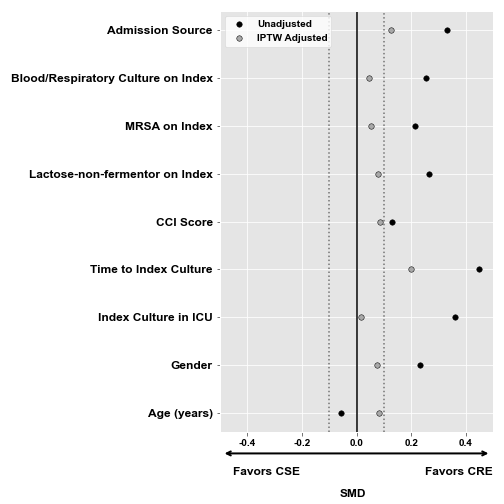 |
| **Abbreviations:** CCI: Charlson Comorbidity Index, IPTW: inverse probability treatment weight, MRSA: methicillin-resistant *Staphylococcus aureus*, SMD: standardized mean difference, ICU: intensive care unit |

## CNSE vs. CSE Main Overall Analysis

| **Supplementary Table 11.** Baseline Balance of Covariates included in Propensity Score Model Before and After IPTW Adjustment for CNSE vs. CSE Overall Analysis | | |
| --- | --- | --- |
| **Variable** | **Unadjusted SMD** | **Adjusted SMD** |
| Age (years) | 0.021 | 0.088 |
| Gender | 0.244 | 0.007 |
| Index Culture in ICU | 0.240 | 0.009 |
| Time to Index Culture | 0.367 | 0.192 |
| CCI Score | 0.118 | 0.050 |
| Lactose-non-fermenting Culture on Index | 0.223 | 0.059 |
| MRSA culture on Index | 0.113 | 0.048 |
| Blood/Respiratory Culture at Index | 0.371 | -0.014 |
| Admission Source | 0.374 | 0.078 |
| **Abbreviations:** CCI: Charlson Comorbidity Index, IPTW: inverse probability treatment weight, MRSA: methicillin-resistant *Staphylococcus aureus*, SMD: standardized mean difference, ICU: intensive care unit.  The standardized mean differences above were measured before and after the application of IPTW-adjustment to ensure balance on the included covariates was achieved. A value of ≤ 0.1 was considered to be sufficiently balanced. | | |

| **Supplementary Figure 6.** Standardized Mean Difference of Covariates included in the Propensity Score Model Before and After IPTW Adjustment for CNSE vs. CSE Overall Analysis |
| --- |
| 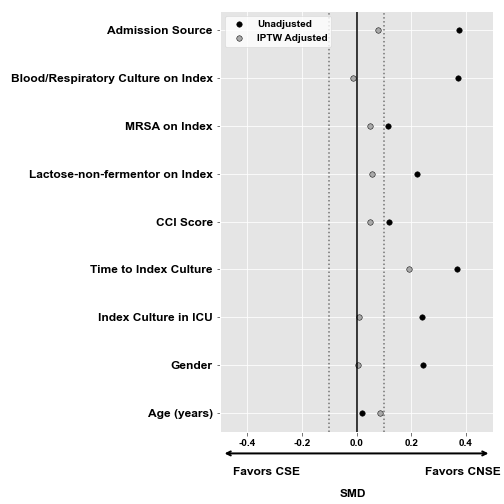 |
| **Abbreviations:** CCI: Charlson Comorbidity Index, IPTW: inverse probability treatment weight, MRSA: methicillin-resistant *Staphylococcus aureus*, SMD: standardized mean difference, ICU: intensive care unit |

## CNSE vs. CSE Bloodstream Infections on Index Date

| **Supplementary Table 12.** Baseline Balance of Covariates included in Propensity Score Model Before and After IPTW Adjustment for CNSE vs. CSE Bloodstream Infection Analysis | | |
| --- | --- | --- |
| **Variable** | **Unadjusted SMD** | **Adjusted SMD** |
| Age (years) | -0.103 | -0.088 |
| Gender | 0.333 | 0.120 |
| Index Culture in ICU | 0.242 | 0.088 |
| Time to Index Culture | 0.205 | 0.115 |
| CCI Score | -0.039 | -0.104 |
| Admission Source | 0.763 | 0.077 |
| **Abbreviations:** CCI: Charlson Comorbidity Index, IPTW: inverse probability treatment weight, MRSA: methicillin-resistant *Staphylococcus aureus*, SMD: standardized mean difference, ICU: intensive care unit.  The standardized mean differences above were measured before and after the application of IPTW-adjustment to ensure balance on the included covariates was achieved. A value of ≤ 0.1 was considered to be sufficiently balanced. | | |

| **Supplementary Figure 7.** Standardized Mean Difference of Covariates included in the Propensity Score Model Before and After IPTW Adjustment for CNSE vs. CSE Bloodstream Infection Analysis |
| --- |
| 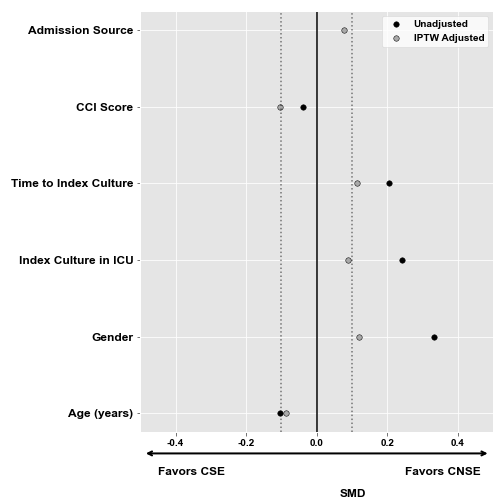 |
| **Abbreviations:** CCI: Charlson Comorbidity Index, IPTW: inverse probability treatment weight, MRSA: methicillin-resistant *Staphylococcus aureus*, SMD: standardized mean difference, ICU: intensive care unit  Note that the unadjusted SMD value for admission source (0.763) is not shown on the figure. The decision was made to leave it off the figure to keep the same units with the other balancing figures. |
